# External validation of the Survival After ROSC in Cardiac Arrest (SARICA) score for predicting survival after return of spontaneous circulation using multinational pan-asian cohorts

**DOI:** 10.3389/fmed.2022.930226

**Published:** 2022-09-08

**Authors:** Maehanyi Frances Rajendram, Faraz Zarisfi, Feng Xie, Nur Shahidah, Pin Pin Pek, Jun Wei Yeo, Benjamin Yong-Qiang Tan, Matthew Ma, Sang Do Shin, Hideharu Tanaka, Marcus Eng Hock Ong, Nan Liu, Andrew Fu Wah Ho

**Affiliations:** ^1^Department of Emergency Medicine, Singapore General Hospital, Singapore, Singapore; ^2^Centre for Quantitative Medicine, Duke-NUS Medical School, Singapore, Singapore; ^3^Pre-hospital and Emergency Research Centre, Duke-NUS Medical School, Singapore, Singapore; ^4^Yong Loo Lin School of Medicine, National University of Singapore, Singapore, Singapore; ^5^Division of Neurology, Department of Medicine, National University Health System, Singapore, Singapore; ^6^Department of Emergency Medicine, National Taiwan University Hospital, National Taiwan University, Taipei City, Taiwan; ^7^Department of Emergency Medicine, School of Medicine, Seoul National University, Seoul, South Korea; ^8^Department of Emergency Medical System, Graduate School of Kokushikan University, Tokyo, Japan; ^9^Health Services and Systems Research, Duke-NUS Medical School, Singapore, Singapore; ^10^SingHealth AI Health Program, Singapore Health Services, Singapore, Singapore

**Keywords:** out-of-hospital cardiac arrest, return of spontaneous circulation, prognosis, survival, resource allocation, emergency department, retrospective cohort study, scoring system

## Abstract

**Aim:**

Accurate and timely prognostication of patients with out-of-hospital cardiac arrest (OHCA) who attain return of spontaneous circulation (ROSC) is crucial in clinical decision-making, resource allocation, and communication with family. A clinical decision tool, Survival After ROSC in Cardiac Arrest (SARICA), was recently developed, showing excellent performance on internal validation. We aimed to externally validate SARICA in multinational cohorts within the Pan-Asian Resuscitation Outcomes Study.

**Materials and methods:**

This was an international, retrospective cohort study of patients who attained ROSC after OHCA in the Asia Pacific between January 2009 and August 2018. Pediatric (age <18 years) and traumatic arrests were excluded. The SARICA score was calculated for each patient. The primary outcome was survival. We used receiver operating characteristics (ROC) analysis to calculate the model performance of the SARICA score in predicting survival. A calibration belt plot was used to assess calibration.

**Results:**

Out of 207,450 cases of OHCA, 24,897 cases from Taiwan, Japan and South Korea were eligible for inclusion. Of this validation cohort, 30.4% survived. The median SARICA score was 4. Area under the ROC curve (AUC) was 0.759 (95% confidence interval, CI 0.753–0.766) for the total population. A higher AUC was observed in subgroups that received bystander CPR (AUC 0.791, 95% CI 0.782–0.801) and of presumed cardiac etiology (AUC 0.790, 95% CI 0.782–0.797). The model was well-calibrated.

**Conclusion:**

This external validation study of SARICA demonstrated high model performance in a multinational Pan-Asian cohort. Further modification and validation in other populations can be performed to assess its readiness for clinical translation.

## Introduction

Out-of-hospital cardiac arrest (OHCA) is a key healthcare challenge for emergency care systems globally, (1) with an estimated incidence of 96 per 100,000 person-years (2). While the pooled incidence of return of spontaneous circulation (ROSC) is 29.7%, only 8.8% achieved 30-day survival globally (3) and 5.8% in the Asia Pacific (4). Advanced interventions post-ROSC can improve mortality in well-selected patients, (5) but also come with significant costs estimated at USD 333,844 per person (6).

The initiation of advanced post-resuscitation efforts often follows after ROSC, in what some authors have described as a “technological imperative” of the physician. Physicians may continue care just because it is available, even if it may not be beneficial to the patient (7). Difficult decisions hence ensue, especially in Asian populations, where such medical decisions often involve the extended family, (8) who might have difficulty comprehending the issue of medical futility under time pressure (9). Accurate prognostication can help frame the family’s expectations and allow for better guidance of decisions, potentially avoiding futile care and facilitating efficient allocation of intensive care resources. However, studies have shown that only 50–70% of physicians are able to accurately predict survival (10). Previous studies have also shown that emergency physicians subjectively terminate resuscitation efforts earlier if there are perceived poor prognostic factors, which may not necessarily be objectively associated with patient outcomes (11). The provision of clear objective clinical decision tools that predict outcomes may hence be used to guide the extent of resuscitative efforts.

A recent systematic review by Gue et al identified several existing OHCA prognostication risk scores with good predictive ability (12). However, the clinical relevance of these scores was noted to be limited by difficulty in computation, recall bias and unavailability of data at the time the patient is in the emergency department (ED). In response to this unmet need, we recently developed the Survival After ROSC In Cardiac Arrest (SARICA) score (13) using real world data from Singapore applied to AutoScore, (14, 15) an interpretable machine learning score generator. SARICA consists of three variables: pre-hospital ROSC, age and initial heart rhythm. On the internal validation cohort, SARICA achieved an area under the curve (AUC) of 0.869 (95% confidence interval 0.839–0.900). There is a pressing need to validate SARICA in external cohorts to further understand its potential for clinical implementation.

In this study, we aimed to externally validate the SARICA score in multinational cohorts within the Pan-Asian Resuscitation Outcomes Study (PAROS).

## Materials and methods

### Study design and setting

We conducted a retrospective cohort study using data from the PAROS registry. PAROS is a clinical research network comprising thirteen countries across the Asia Pacific, and collects out-of-hospital cardiac arrest data. Participating communities are required to submit all core variables regarding each arrest (including bystander CPR, out-of-hospital defibrillation, ROSC in the ED), including information from both Emergency Medical Services and participating hospitals (4). Communities with existing cardiac arrest registries contributed data *via* an export field entry process into the PAROS registry. All data was further verified by designated coordinators in each participating community. Further checks were performed by the trial coordinating center that ensured clarification of logical inconsistencies and missing data through source verification. Further information regarding data collection has been previously described (4).

### Participants

We included all OHCA cases between January 2009 and August 2018 that attained ROSC. OHCA was defined as the absence of pulse, unresponsiveness, and apnea; ROSC was defined as regaining a palpable pulse. Cases that were not attended to by Emergency Medical Services (EMS) were excluded. Countries with fewer than 500 patients who attained ROSC were excluded due to small effect size. Data from Singapore was excluded as that data had previously formed the derivation and internal validation cohorts (13). Pediatric arrests (age <18 years) and traumatic arrests were excluded. Cases with missing data (of key variables required in computation of the SARICA score, and the primary outcome) were excluded. This study was approved by SingHealth Centralised Institutional Review Board (CIRB ref: 2013/604/C) and Domain Specific Review Board (ref: C/10/545 and 2013/00929) with waiver of informed consent.

### Calculation of survival after ROSC in cardiac arrest score

The SARICA score was calculated as described in the original SARICA paper (13). SARICA comprises three variables: age, pre-hospital ROSC and initial shockable rhythm. For age, in years, it awarded 0 points for age ≥80, 1 point for age 60 to 79, 2 points for age 40 to 59 and 3 points for age <40. Pre-hospital ROSC was awarded 4 points, and initial shockable rhythm was awarded 3 points. The sum of the scores from each variable formed the SARICA score, with the total score ranging from 0 to 10 points.

### Outcomes

The primary outcome was survival, which was defined as survival to hospital discharge or being alive in hospital at 30 days. Secondary outcome was good neurological recovery at 30 days post-arrest, defined as Glasgow-Pittsburgh cerebral performance category (CPC) scores 1 to 2.

### Statistical methods

Data preparation, descriptive analysis, and receiver operating curve (ROC) analysis were performed using IBM SPSS Statistics 25.0 (Armonk, NY, United States). Descriptive statistics were generated to compare the characteristics of survivors and non-survivors. Data was reported as mean and standard deviation (SD) for continuous variables and percentages for categorical variables. Bivariable analysis by survival was performed with a chi-squared test for categorical variables, and independent samples *t*-test for continuous variables. We performed ROC analysis on the overall cohort, and subsequently on pre-determined subgroups. Sensitivity and specificity were calculated for each of the SARICA scores. A calibration belt plot was then constructed to assess model calibration.

This study did not venture to identify a threshold score as it is outside the scope of our study; clinical application of such a scoring would be highly dependent on the population it is applied to, as determining distribution of resources based on predicted mortality would depend highly on resource availability. Instead, we aim to validate the performance of the original SARICA score, which similarly, did not propose any cut-off.

## Results

**FIGURE 1:**
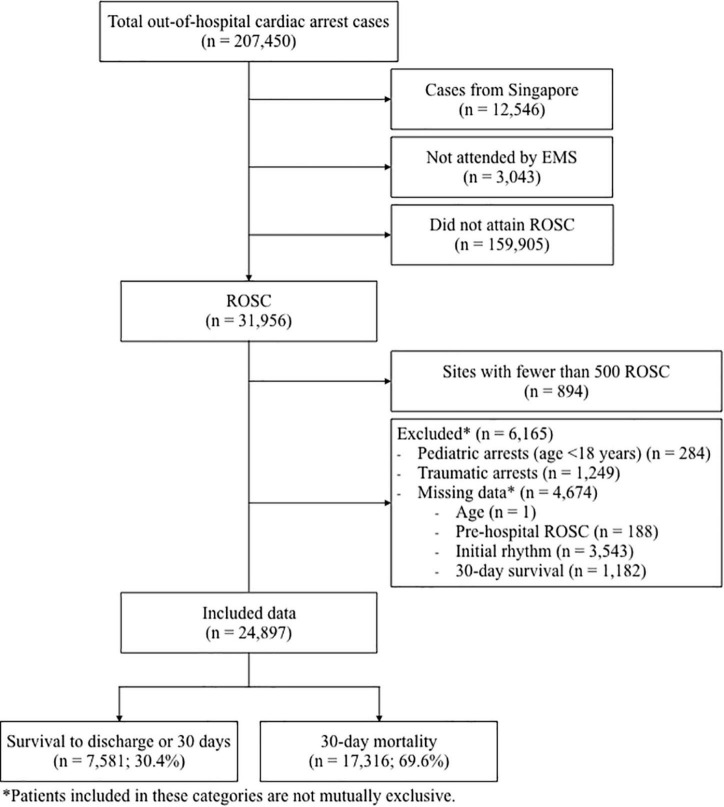
Population flow diagram showing cohort selection. EMS, emergency medical services; ROSC, return of spontaneous circulation.

There was a total of 207,450 cases of OHCA reported to the PAROS registry from January 2009 to August 2018. A total of 12,546 cases from Singapore, 3,043 that were not attended by EMS and 159,905 cases that did not attain ROSC, were excluded. Of the 31,956 who did attain ROSC, 6,165 cases (3% of total population) were excluded due to missing data. Of the 12 remaining countries in the PAROS registry, 9 countries (with a total of 894 cases) were further excluded as they each had fewer than 500 patients with ROSC. The remaining countries included in the analysis were Japan, South Korea and Taiwan. Finally, 24,897 cases qualified for analysis. The population flow diagram in Figure 1 demonstrates the selection of study participants.

### Characteristics of study population

The clinical characteristics of the study cohort, along with comparisons of survivors vs. non-survivors, are shown in Table 1. The cohort had a mean age of 69.3 (SD 16.0) years, and 64.1% were male. A total of 7,581 patients (30.4%) survived to hospital discharge or 30 days. Survivors, compared to non-survivors, were younger (64.2 vs. 71.6 years old, *p* < 0.001) and more likely to be male (70.5 vs. 61.2%, *p* < 0.001). Compared to non-survivors, a greater proportion of survivors had a witnessed arrest (79.7 vs. 64.3%, *p* < 0.001), bystander cardiopulmonary resuscitation (CPR) (46.0 vs. 41.4%, *p* < 0.001), and bystander automated external defibrillator (AED) use (3.7 vs. 2.1%, *p* < 0.001). Significantly more survivors had an initial shockable rhythm compared to non-survivors (47.2 vs. 14.1%, *p* < 0.001), and received pre-hospital defibrillation (51.5 vs. 19.3%, *p* < 0.001). A lower proportion of survivors received pre-hospital drug administration (13.3 vs. 22.5%, *p* < 0.001) and insertion of advanced airway (24.1 vs. 49.9%, *p* < 0.001). A total of 73.2% of survivors had pre-hospital ROSC as compared to 40.9% of non-survivors (*p* < 0.001).

**TABLE 1:** Clinical characteristics of the study cohort, with comparison between survivors and non-survivors.

	All, *n* (%)	Survivors, *n* (%)	Non-survivors, *n* (%)	*P*-value
				
Total	24,897	7,581 (30.4%)	17,315 (69.5%)	–
Gender, male	15,948 (64.1%)	5,348 (70.5%)	10,600 (61.2%)	<0.001
Age in years, mean	69.3 ± 16.0	64.2 ± 16.3	71.6 ± 15.3	<0.001
Age in years				<0.001
<40	1,290 (5.2%)	626 (8.3%)	664 (3.8%)	
40 to 59	4,946 (19.9%)	2,081 (27.5%)	2,865 (16.5%)	
60 to 79	11,000 (44.2%)	3,458 (45.6%)	7,542 (43.6%)	
≥80	7,661 (30.8%)	1,416 (18.7%)	6,245 (36.1%)	
**Past medical history**				
Heart disease	3,844 (29.6%)	1,052 (32.1%)	2,792 (28.7%)	<0.001
Diabetes mellitus	2,418 (31.5%)	543 (26.5%)	1,875 (33.3%)	<0.001
Hypertension	3,632 (46.9%)	887 (42.8%)	2,745 (48.4%)	<0.001
Hyperlipidemia	227 (3.3%)	83 (4.5%)	144 (2.9%)	<0.001
Renal disease	729 (10.6%)	169 (9.2%)	560 (11.1%)	<0.001
Respiratory disease	630 (9.1%)	145 (7.9%)	485 (9.6%)	<0.001
Stroke	867 (12.9%)	177 (9.6%)	690 (13.5%)	<0.001
Cancer	881 (12.6%)	139 (7.5%)	742 (14.5%)	<0.001
**Details of arrest**				
Witnessed arrest	16,596 (69.%)	5,893 (79.7%)	10,703 (64.3%)	<0.001
Bystander CPR	10,633 (42.8%)	3,480 (46.0%)	7,153 (41.4%)	<0.001
Bystander AED use	399 (2.6%)	190 (3.7%)	209 (2.1%)	<0.001
Initial shockable rhythm	6,017 (24.2%)	3,582 (47.2%)	2,435 (14.1%)	<0.001
Pre-hospital defibrillation	7,252 (29.1%)	3,906 (51.5%)	2,246 (19.3%)	<0.001
Pre-hospital advanced airway inserted	11,228 (45.7%)	2,709 (24.1%)	8,519 (49.9%)	<0.001
Pre-hospital drug administered	4,862 (19.7%)	1,001 (13.3%)	3,861 (22.5%)	<0.001
Pre-hospital adrenaline given	4,822 (25.5%)	983 (16.3%)	3,839 (29.8%)	<0.001
Pre-hospital ROSC	12,639 (50.8%)	5,552 (73.2%)	7,087 (40.9%)	<0.001

ROSC, return of spontaneous circulation; CPR, cardiopulmonary resuscitation; AED, automated external defibrillator.

### Calculated survival after ROSC in cardiac arrest score for study population

The median SARICA score was 4 (IQR 1-5). The proportion of patients who survived or had a good neurological outcome by each SARICA score level is shown in Table 2. There was a monotonic relationship between SARICA score and proportion of survivors. There was also a visible positive correlation with good neurological outcome; only 1.4% of patients with SARICA score 0 survived with good neurological outcome, while 74.1% of patients at SARICA score 10 survived with good neurological outcome.

**TABLE 2:** Distribution of clinical outcomes by SARICA score level, along with sensitivity and specificity for each cut-off.

Score cut-off	Proportion of study population (%)	Proportion of survivors within the score (%)	Proportion of survivors with good neurological outcome within the score (%)	Sensitivity (%)	Specificity (%)
≥0	100	9.2	1.4	100	0.0
≥1	85.9	13.6	2.8	95.7	18.4
≥2	67.7	17.2	3.2	87.6	41.1
≥3	60.1	19.0	3.4	83.3	50.1
≥4	57.5	25.5	9.9	81.7	53.1
≥5	40.5	35.2	17.4	67.4	71.3
≥6	23.2	41.5	26.0	47.5	87.4
≥7	18.0	43.5	25.0	40.3	91.8
≥8	14.5	68.4	50.9	35.4	94.6
≥9	6.3	80.5	67.0	17.1	98.3
≥10	1.4	86.8	74.1	4.0	99.7

### Score validation

**FIGURE 2:**
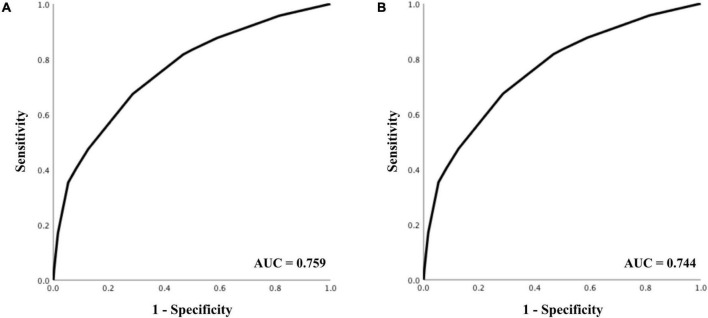
Receiver operating characteristics analysis for prediction of **(A)** 30-day survival, and **(B)** good neurological outcome.

The AUC for predicting survival was 0.759 (95% CI 0.753–0.766), indicating acceptable diagnostic accuracy. AUC for predicting good neurological outcome was 0.744 (95% CI 0.732–0.755), which was also acceptable. The respective receiver operating characteristics (ROC) curves can be seen in Figure 2.

Receiver operating characteristics analysis on predetermined subgroups showed acceptable diagnostic accuracy across most subgroups in predicting survival (see Table 3). The score exhibited reduced diagnostic accuracy in two subgroups–administration of adrenaline pre-hospital (AUC 0.652), and presumed respiratory etiology of cardiac arrest (AUC 0.630), but improved diagnostic accuracy in subgroups that received bystander CPR (AUC 0.791) and of presumed cardiac etiology (AUC 0.790).

**TABLE 3:** Subgroup analysis–survival rate, area under the curve.

Subgroup	Survival (%)	AUC (95% CI)
Total cohort	30.4%	0.759 (0.753–0.766)
Subgroups by site		
Japan	33.2%	0.759 (0.751–0.767)
Korea	26.0%	0.771 (0.756–0.786)
Taiwan	26.4%	0.695 (0.676–0.715)
Witnessed arrest	35.5%	0.754 (0.747–0.762)
Unwitnessed arrest	20.2%	0.740 (0.725–0.754)
Bystander CPR	32.7%	0.791 (0.782–0.801)
Bystander AED	47.6%	0.746 (0.698–0.793)
Pre-hospital defibrillation	53.9%	0.753 (0.741–0.764)
Pre-hospital airway	24.1%	0.731 (0.720–0.743)
Pre-hospital drug administration	20.6%	0.654 (0.633–0.674)
Pre-hospital adrenaline	20.4%	0.652 (0.632–0.672)
Defibrillation in ED	24.7%	0.750 (0.723–0.777)
Advanced airway inserted in ED	23.6%	0.746 (0.729–0.763)
Presumed cardiac etiology	33.1%	0.790 (0.782–0.797)
Presumed respiratory etiology	31.4%	0.630 (0.602–0.658)

### Calibration performance

**FIGURE 3:**
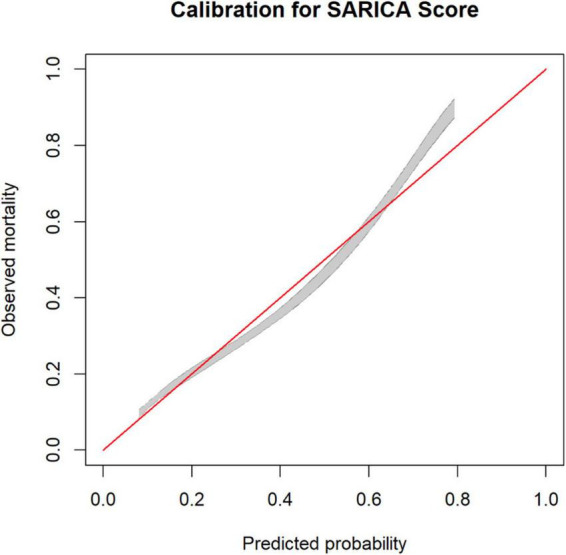
Calibration plot for SARICA score showing calibration belts (at 80% [gray] and 95% [dark gray] confidence levels).

A calibration belt plot was used to plot observed outcome vs. predicted probability (Figure 3). The calibration was deemed very good as the calibration belt approximated the line *y* = x.

## Discussion

This external validation study of SARICA in a multinational Pan-Asian cohort demonstrated good model performance (both accuracy and calibration). SARICA exhibited reduced diagnostic accuracy among patients who received pre-hospital adrenaline and those of respiratory etiology. It exhibited improved diagnostic accuracy among patients who received bystander CPR and those of presumed cardiac etiology. This is the first external validation study of the SARICA score.

Several other scores have been created as prognostic tools for patients after ROSC, including NULL-PLEASE, OHCA, and rCAST, each of which exhibit good predictive value (AUC >0.8) (12). However, these scores require variables not immediately attainable on arrival to the emergency department (e.g., serum lactate, pH level, cause of arrest) or variables that are subject to recall bias (e.g., duration of low-flow or no-flow time), or require complex calculations and a score calculator for interpretation. SARICA employs three easily obtainable, routinely available, and objective variables (age, initial shockable rhythm, and pre-hospital ROSC) to offer accurate prediction of prognosis–fulfilling an unmet clinical need in accurate and timely prognostication in the emergency department.

The AUC in our external validation study is lower than that of the internal validation study (AUC 0.759 as compared to 0.869). This appears to be a common feature in prediction scores for OHCA, including the OHCA, (16) NULL-PLEASE (17), and CAHP (18) scores. A possible reason could be the heterogeneity in different healthcare systems–including differences in ambulance arrival time, prevalence and use of AEDs, expertise of first responders–which can contribute to varying outcomes. There was inadequate data for subgroup analysis for evaluation of these possible confounders.

Differences in cultural attitudes toward life-sustaining treatment (LST) can also confound outcomes and contribute to the variation in survival rate between populations at the same SARICA level.

Withholding of LST is prevalent in East Asia, and can contribute to falsely low survival rates. A study by Phua et al. (19) revealed that 70% of physicians in Asian countries would almost always or often withhold LST, and 82% would implement do-not-resuscitate orders, for patients with no real chance of recovering a meaningful life. Interpretation and selection of this patient group remains highly subjective, and can result in limitation of care for patients with perceived poor prognosis, resulting in a falsely low survival rate. This may result in a self-fulfilling prophecy where patients of presumed poor prognosis are denied medical care, thereby decreasing survival rates.

On the other hand, withdrawal of LST, or lack thereof, can also confound survival rates. Among the 3 studied populations, only Taiwan permits withdrawal of LST with persistent vegetative state (20). Korea only allows withdrawal of LST in patients who are imminently dying, (21) while Japan has no official law regarding withdrawal of LST, with a previous survey showing that physicians’ fear of criminal prosecution has contributed to avoidance of withdrawal of LST (22). Asian families also play an important role in medical decision making, (23) and these decisions often lean toward prolonging life (24).

At SARICA score 0 to 2, our study population demonstrates higher survival rates, but 80% of these patients are of poor neurological outcome. This may suggest decreased rates of withdrawal of life-sustaining treatment (LST) despite poor neurological recovery. A total of 71 to 80% of ICU physicians in Japan, Taiwan and Korea believe that withholding care and withdrawing care are ethically dissimilar, compared to 41% in Singapore, the original study population for SARICA (19). This mindset that withdrawing LST is ethically unacceptable can contribute to high survival rates despite poor neurological outcomes at low SARICA scores.

Nevertheless, an AUC of 0.759 with good calibration indicates a respectable predictive accuracy. Within our study, other factors that demonstrated good correlation with survival were pre-hospital defibrillation (OR 4.44, 95% CI 4.18–4.71), witnessed arrest (OR 2.18, 95% CI 2.04–2.32) and public location of arrest (OR 2.40, 95% CI 2.26–2.55). These factors have been proven to correlate with eventual survival (4, 25) and are also included in other predictive scores such as NULL-PLEASE, (26) CaRdiac Arrest Survival Score, (27) and Cardiac Arrest Hospital Prognosis score (28). They also remain in line with our aim of employing objective variables that are easily obtained in the emergency department, without being subject to recall bias. However, their inclusion did not substantially improve overall model performance (measured by AUC) as shown in the parsimony plot of the original SARICA derivation paper (13).

Moving forward, despite good model performance, clinical implementation of the SARICA score remains limited at present. Clinical scoring systems require a specific cut-off to guide clinical decision making, however, identifying a specific cut-off remains beyond the scope of our study. Firstly, a sensible cut-off for one setting may be irrelevant for another. A sensible cut-off is one that rations life-sustaining resources (including intensive care unit beds, among others) rationally, which would depend highly on the availability of resources. A healthcare system that faces severe resource limitation may hence be compelled to accept a higher specificity to reduce its false-positive rate, in order to conserve scarce resources. Secondly, prior to recommending a cut-off point, there is a necessary step of determining how many levels the score should have. The original derivation study arbitrarily used a 10-point scale, however, it could be that a 20-point scale is required to produce the sensitivity and specificity desired.

### Limitations

There are several limitations of our study. First, similar to cohort selection in the original SARICA derivation publication, (13) we excluded cases that were not attended by EMS, traumatic arrests, and pediatric cases. These may limit the generalizability of our results to these subgroups of OHCA patients. However, we note that these collectively comprised only 2% of all cases. The clinical implementation process of SARICA would therefore require education of clinicians on patient groups on which SARICA lacked robust validation data so far. Second, we had to exclude cases that had missing data for any of the three variables required to compute SARICA. The proportion of missing data varied across sites. However, it is unlikely that the missing data would skew overall survival, hence we believe the robustness of our analysis is not in question. Third, neurological recovery was assessed through CPC score at 30 days post-arrest. As patients may progress in their neurological recovery beyond this point, eventual neurological outcome may not be accurately reflected. Evaluation of CPC score also requires assessment of community-level functioning that is difficult to perform while inpatient, and may further affect accuracy of the assessment. Lastly, a strong cultural influence on withdrawal and withholding of life support in East Asia also confounds outcomes–clinicians are averse toward withdrawal of LST, resulting in higher rates of survival with poor neurological outcome, but are inclined toward withholding of LST, where a self-fulfilling prophecy of perceived poor outcome may result in inadequate escalation of care and falsely low survival rates.

## Conclusion

This external validation study of SARICA demonstrated high model performance (AUC 0.759) in a multinational Pan-Asian cohort. However, further validation is required before clinical application. This can include further increasing the number of score levels to create a score with higher specificity and a lower false positive rate. Further analysis to determine a threshold score and additional validation in populations outside East Asia will also aid in improving the score for clinical application.

## Membership of the pan-asian resuscitation outcomes study clinical research network participating site investigators

AlQahtani S (National Ambulance, Abu Dhabi, United Arab Emirates); BSH Leong (National University Hospital, Singapore, Singapore); Cai W (Zhejiang Provincial People’s Hospital, Zhejiang, China); CH Lin (National Cheng Kung University, Tainan, Taiwan); CW Kuo (Chang-Gung Memorial Hospital, Taoyuan City, Taiwan); DN Son (Bach Mai Hospital, Hanoi, Vietnam); FJ Gaerlan (Southern Philippines Medical Center, Davao City, Philippines); HN Gan (Changi General Hospital, Singapore, Singapore); HW Ryoo (Kyungpook National University, Daegu, South Korea); K Kajino (Kansai Medical University Hospital, Osaka, Japan); K Sarah (Hospital Sungai Buloh, Selangor, Malaysia); Khan N (Aga Khan University Hospital, Karachi, Pakistan); Ko PCI (National Taiwan University, Taipei, Taiwan); L Tiah (Changi General Hospital, Singapore, Singapore); Mao DRH (Khoo Teck Puat Hospital, Singapore, Singapore); MYC Chia (Tan Tock Seng Hospital, Singapore, Singapore); NE Doctor (Sengkang General Hospital, Singapore, Singapore); Ng YY (Tan Tock Seng Hospital, Singapore, Singapore); Nguyen DA (Bach Mai Hospital, Hanoi, Vietnam); P Khruekarnchana (Rajavithi Hospital, Bangkok, Thailand); Rao R (GVK Emergency Management and Research Institute, Telangana, India); RH Ho (Chonnam National University Medical School and Hospital, Gwangju, South Korea); S Arulanandam (Singapore Civil Defence Force, Singapore, Singapore); SO Cheah (Urgent Care Clinic International, Singapore, Singapore); Supasaowapak J (Rajavithi Hospital, Bangkok, Thailand); Tagami T (Nippon Medical School Tama Nagayama Hospital, Tokyo, Japan); Velasco B (East Avenue Medical Center, Manila, Philippines); Vimal M (GVK Emergency Management and Research Institute, Telangana, India); WM Ng (Ng Teng Fong General Hospital, Singapore, Singapore); Wong KD (Hospital Pulau Pinang, Penang, Malaysia); Zhou SA (Zhejiang Provincial People’s Hospital, Zhejiang, China).

## Data availability statement

The raw data supporting the conclusions of this article will be made available by the authors, without undue reservation.

## Ethics statement

The studies involving human participants were reviewed and approved by the SingHealth Centralised Institutional Review Board Domain Specific Review Board. Written informed consent for participation was not required for this study in accordance with the national legislation and the institutional requirements.

## Author contributions

MR, FZ, NS, NL, and AH contributed to conception and design of the study. MR, FX, NS, PP, MM, SD, HT, MO, NL, and AH were involved in data curation. MR and FX performed the statistical analysis. MR, FX, and AH wrote the first draft of the manuscript. All authors contributed to the manuscript revision, read, and approved the submitted version.
